# On the Quantum and Tempo of Women First Marriages in China

**DOI:** 10.3390/ijerph192013312

**Published:** 2022-10-15

**Authors:** Jingyi Dan, Nong Zhu, Li Mei

**Affiliations:** 1 Institute for Population and Development Studies, School of Public Policy and Administration, Xi’an Jiaotong University, Xi’an 710049, China; 2 INRS—Urbanization Culture Society Research Centre, Montreal, QC H2X 1E3, Canada

**Keywords:** total first marriage rate, tempo-adjusted period proportion ever married, quantum change, tempo effects, decomposition

## Abstract

The total first marriage rate (TFMR) of Chinese women shows a downward trend in fluctuations from 1970 to 2016, but it is affected by the tempo distortion caused by changes in the mean age at first marriage. Thus, we compare the total first marriage rate (TFMR) and tempo-adjusted period proportion ever married (PPEM*) to estimate the extent to which the TFMR is affected by tempo effects. We also decompose the women’s TFMR change into its quantum and tempo components from 1970 to 2016 to analyze how much of the changes in TFMR are due to the quantum changes and how much of it is caused by tempo effects. The results show that the tempo effects have had a persistent influence on the period TFMR of Chinese women from 1970 to 2016. The recent decline in the TFMR in Chinese women is mainly due to the first marriage delay, not signaling a retreat from universal marriage.

## 1. Introduction

The marriage pattern among Chinese women is characterized by two phenomena—early marriage and universal marriage [1,2]. In historical Chinese society, women typically married at a very early age, and only a tiny proportion of women were never married [3,4]. This was underscored by strong patrilineal values in a form where marriage existed to perpetuate family lines as well as strong patriarchal values in a form where marriage served as an avenue of social and economic security for women [5,6]. In recent years, China has undergone a series of socio-economic development which were not only rapid but also agents of ideological change.

The education expansion, economic growth, and women’s economic participation have enhanced women’s employment opportunities and financial independence [7]. Thus, the hitherto reliance of women on marriage for economic security has relatively weakened. The extended effect has also manifested in the recent trend of delayed first marriage [8,9]. On the extreme side is the ideology of “leftover women”(sheng nü), which is a somewhat derogatory word used to refer to women who are unmarried, highly educated and over 27 years [10].

Demographers usually analyze total first marriage rate (TFMR) to monitor marriage trends and level. As a form of period measure, the TFMR describes the proportion of women (or men) in a birth cohort who would become ever-married if that cohort experienced an age-specific first marriage ratio prevailing among women (or men) in a given year [11]. The cohort in this context is a hypothetical measure, allowing for a cohort-like interpretation. When all women in a cohort (100%) enter first marriage, the TFMR of that cohort would equal 1.00. In China, there is a universal first marriage for women. Universal marriage is sometimes defined as fewer than 5% of women remaining single at the age of 50.00 [12,13]. So, with a marriage proportion of greater than 95%, the TFMR of Chinese women should be very close to 1.00.

Several factors have directly or indirectly contributed to changes in the trend of TFMR in China over the years. From 1950 to 1979, the TFMR fluctuated, sometimes above 1.00, but for the most part, it was below 1.00. Nevertheless, a decline started in the early 1970s. The legal marriage age in the 1950 marriage law stipulated 20.00 and 18.00 years for males and females, respectively [14]. In the early 1970s, the government introduced the “later, longer, and fewer” policy. The policy encouraged young people to delay marriage until age 25.00 and 23.00 years for males and females, respectively [15]. This caused women’s mean age at first marriage to increase rapidly during the 1970s [16,17,18], thus declining the TFMR [4]. After a new marriage law was implemented in 1981, the TFMR increased in the 1980s [19]. The change is attributed to the fact that with the 1981 marriage law, the age requirement for marriage had been lowered compared to the workings of the “later, longer, and fewer” policy, causing the mean age at first marriage of women to fall briefly [18,20]. Since 1990, women’s mean age at first marriage has consistently risen, which is a direct effect of the economic growth and education expansion at that time [21,22]. According to the 2020 census data, women’s mean age at first marriage has reached 27.95 years, and given the inverse relationship between TFMR and mean age at first marriage, the TFMR has declined further in recent years [23].

The TFMR consists of quantum and tempo components. The quantum component equals the proportion of the people who marry in the absence of changes in the timing of first marriage during the period. The tempo component is equal to the distortion that occurs due to first marriage timing changes [24]. Specifically, if the women shift the ages at which they enter first marriage later without changing their completed first marriage rates, annual numbers of first marriages will be less than they would have been because the same number of first marriages will be spread out over a longer time period. Similarly, if women enter first marriages at younger ages, the annual numbers of first marriages will be larger than they would have been because the same number of first marriages occurs over a shorter time period. These changes in the annual number of first marriages induced by changes in the timing of first marriages are tempo effects [25]. In other words, when the mean age at first marriage rises, the TFMR declines, and vice versa when the mean age at first marriage declines—an inverse relationship.

Due to the shifts in women’s mean age at first marriage (changes in marriage timing), the TFMR is often over or underestimated. For instance, the TFMR was as low as 0.74 in 1977 [19]. It may be erroneous to conclude that the marriage level in 1977 implied that only 74% of Chinese women eventually married. In addition, after the 1981 marriage law, the TFMR increased to 1.23 in 1982 [19]. This may indicate an anomaly because, ideally, women can only experience at most one first marriage. Such an anomaly that results from changes in marriage timing is called tempo distortion [24]. Therefore, from the foregoing, the recent decline in the TFMR in China might simply be due to changes in age at first marriage; it might not actually signal a retreat from universal marriage.

In order to improve readings on period first marriage level and trends, scholars have proposed methods for adjusting and removing tempo distortion in TFMR. Among these have included the tempo-adjusted total first marriage rate (TFMR*), the period proportion ever married (PPEM), and its tempo adjustment variant, PPEM* [24].

Previous researchers have analyzed the quantum and tempo effects of TFMR and PPEM. For instance, Winkler-Dworak and Engelhardt [26] explore how much of the change in women’s first marriage rates in Austria, Germany, and Switzerland can be attributed to tempo or quantum effects. Their result showed that a significant share of the decline in first marriage rates was due to tempo distortions in all three countries, although in varying degrees. Schoen and Canudas-Romo [27] used a variant of the Timing Index developed in research on fertility to measure cohort timing effects for marriage and calculate an adjusted period proportion ever marrying in England, Wales, and the USA. They find substantial tempo effects on the period proportion ever marrying, and the adjusted period proportion ever marrying values show a real decline in marriage for cohorts.

In the context of China, studies on TFMR’s quantum or tempo effects are limited. In their analysis of the marriage squeeze in China, Jiang et al. [28] normalized first marriage frequency by eliminating the tempo effect in first marriage. In a much older study, Coale et al. [4] analyzed the trend in marriage and fertility between 1950 and 1981, and their result suggested that the TFMR is affected by tempo effects: that is, changes in the mean age at first marriage. Given the recent changes in the mean age at first marriage, it is vital to analyze the trends of women’s TFMR in recent years and how much of the changes in the TFMR are due to the quantum changes, and how much of it is caused by tempo effects. Based on this, this study is designed to analyze the trend of TFMR between 1970 and 2016. Using decomposition analysis, changes in the TFMR during this period will be decomposed into quantum and tempo effects.

## 2. Methods

### 2.1. The Total First Marriage Rate (TFMR)

The primary focus of this study is to describe the current marriage level among Chinese women. The age of first marriage is set between 15 and 49. While this age bracket is the default selection for fertility, also in demography, a person who is never married by age 50 is regarded as lifelong never married, and first marriage rate is very low among Chinese women under age 15: hence, the adoption of the 15–49 age range for analyzing first marriage. The TFMR is estimated as the sum of the age-specific rate of the second kind (incidence rates) of first marriage [24]. It can be written as:

(1) $$TFMR(t)=\sum_{x=15}^{49}\frac{FM_{x,t}}{{\bar{P}}_{x,t}}$$

where $FM_{x,t}$ indicates the number of first marriages of women aged *x* during year *t*. ${\bar{P}}_{x,t}$ indicates the average populations of women aged *x* at the beginning and end of the reference year *t*.

However, while this indicator has been widely used, it is susceptible to tempo distortion, which would cause the TFMR to exceed 1.00. Researchers have proposed several methods to assuage this shortcoming in calculating the indicator. Among the methods is the tempo-adjusted period proportion ever married (PPEM*), which will be utilized in the current analysis. The core advantage of the PPEM* is that its values are restricted to one by definition based on the nuptiality table, and produce more stable values, as opposed to the TFMR*, which often displays large fluctuations [24].

### 2.2. The Tempo-Adjusted Period Proportion Ever Married (PPEM*)

The PPEM* was introduced by Bongaarts and Feeney [24] to adjust for tempo distortion in calculating age-specific rates of first kind (occurrence/exposure rates) of first marriage from the nuptiality table. It can be written as:

(2) $$PPEM^{*}(t)=1-\exp[-\sum_{x=15}^{49}\frac{FM_{x,t}/{\bar{UM}}_{x,t}}{1-r(t)}]$$

where the ${\bar{UM}}_{x,t}$ indicates the average population of never-married women aged *x* at the beginning and end of the reference year *t*. r(t) indicates the annual rate of change in the mean age at first marriage. Originally, the period tempo-adjusted is adapted from the analysis of fertility [29]. In the field of fertility, the tempo-adjusted indicator is used to remove the distortion effect influencing the total fertility rate (TFR) [30,31]. Compared to the field of marriage, the tempo-adjusted indicator in fertility rate is analogous to the tempo-adjusted indicator in the marriage rate. That is, we can compare cohort first marriage rates and PPEM* to illustrate that the PPEM* performs well in removing tempo distortions.

Based on the Bongaarts and Sobotka method[30,31], we use the data from the 2005 China 1% population sample survey to calculate the cohort first marriage rates and the cohort mean age at first marriage of women cohorts born in 1950 to 1956. The cohort born year (1950–1956) plus mean age at first marriage of these cohorts leads to the years 1972, 1973, 1974, 1976, 1977, 1978, and 1979. Then, the PPEM* for each of these years was compared with the corresponding cohort first marriage rates. The results of the PPEM* and cohort first marriage rate are very close. PPEM* is therefore the preferred indicator for the analysis of tempo distortions.

### 2.3. Measuring Tempo Distortions and Decomposition Analysis

As mentioned above, the PPEM* aims to measure the period quantum of total first marriage rate that is free from tempo distortions. Therefore, the difference between the TFMR and PPEM* measures the tempo distortions in the TFMR, and it can be written as:

(3) $$\mathrm{tempo}\;\mathrm{effect}=TFMR(t)-PPEM^{*}(t)$$

The change in TFMR consists of a quantum change and tempo effects. Based on the method of Yoo and Sobotka [32], we decomposed the change of TFMR into quantum change and the change caused by tempo effect, using the following analysis:

(4) $$\begin{array}{l} TFMR(t_{2})-TFMR(t_{1})\\ =TFMR(t_{2})-TFMR(t_{1})+PPEM^{*}(t_{2})-PPEM^{*}(t_{2})+PPEM^{*}(t_{1})-PPEM^{*}(t_{1})\\ =[PPEM^{*}(t_{2})-PPEM^{*}(t_{1})]\\ +\{[TFMR(t_{2})-PPEM^{*}(t_{2})]-[TFMR(t_{1})-PPEM^{*}(t_{1})]\} \end{array}$$

Here, ($\left[PPEM^{*}(t_{2})-PPEM^{*}(t_{1})\right]$) represents the quantum change. $\{[TFMR(t_{2})-PPEM^{*}(t_{2})]-[TFMR(t_{1})-PPEM^{*}(t_{1})]\}$ represents change caused by the tempo effects.

## 3. Data

Data for this study were obtained from the 2005 China 1% Population Sample Survey and the 2017 China Fertility Sample Survey. China’s National Bureau of Statistics and The National Health and Family Planning Commission (now renamed the National Health Commission) conducted both surveys, respectively. These two surveys are nationally representative and included samples from the 31 provinces, municipalities, and autonomous regions in mainland China. In both surveys, part of the information collected includes the date of birth, date of the first marriage, and marital status of women including those aged 15–60. The first two were used to generate age at first marriage. The sample size includes 1,288,199 and 243,951 women from the 2005 and 2017 surveys, respectively. After the exclusion of observations with missing values in marital status of women (19.46%) in the 2005 survey, the final sample included for analysis was 1,037,578 and 243,951 women from the 2005 and 2017 surveys, respectively. Based on these data, we obtained the number of first marriages of women, the number of never-married women, and the total number of women, and then calculated women’s TFMR and PPEM* from 1970 to 2016. Results from 1970 to 2004 were calculated from the 2005 China 1% Population Sample Survey, and the results from 2005 to 2016 were calculated from the 2017 China Fertility Sample Survey.

## 4. Results

### 4.1. TFMR and PPEM* Trends, 1970–2016

**Figure 1:**
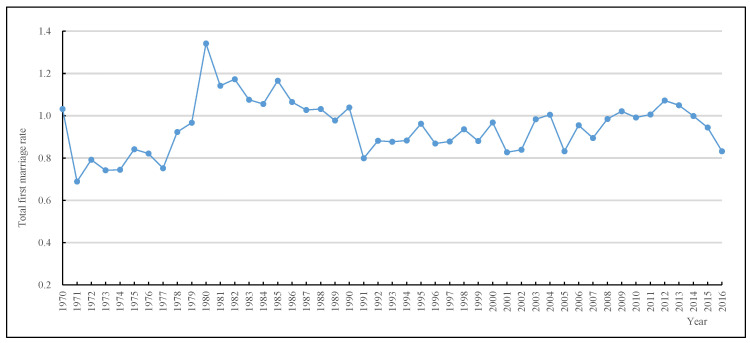
Total first marriage rate trends of Chinese women from 1970 to 2016. Source: 2005 China 1% Population Sample Survey and the 2017 China Fertility Sample Survey.

Figure 1 shows the women’s TFMR trends in China between 1970 and 2016. The TFMR showed a downward trend in fluctuations from 1970 to 2016. Between 1970 and 1979, the TFMR was below 1.00; for the most part, it stayed at a relatively low level, the lowest falling to 0.69 in 1971. At the beginning of the 1980s, the TFMR had already climbed higher and was above 1.00. However, it declined rapidly from 1980 to 1990, especially in the early to mid-1980s. From 1990 to 2007, the TFMR was lower but broadly stabilized with little fluctuations compared to the 1980s—it remained in the range of 0.80–1.00. Since 2007, the TFMR showed an upward trend, reaching the highest value of 1.07 in 2012. After that, it went downward, falling to 0.83 in 2016, which was the lowest value in a decade.

**Figure 2:**
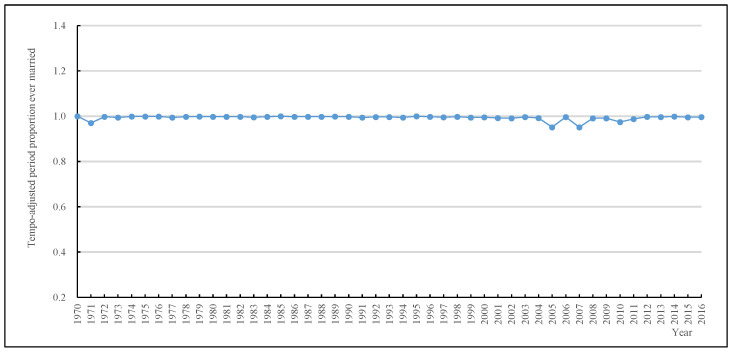
Tempo-adjusted period proportion ever married trends of Chinese women from 1970 to 2016. Source: 2005 China 1% Population Sample Survey and the 2017 China Fertility Sample Survey.

Figure 2 shows the trends of women’s PPEM* in China from 1970 to 2016. The PPEM* consistently maintained a high level throughout the analyzed period, and the values fluctuated between 0.95 and 1.00. It is worth noting that the PPEM* gives smoother and more stable trends over time than the TFMR at all times. Comparing the trends of the TFMR and the PPEM*, the value of the TFMR was significantly greater than the PPEM* in the 1980s, and the value of the TFMR was less than the PPEM* in nearly all other years, especially in the 1970s. It indicates that the TFMR is affected by changes in timing of first marriage.

### 4.2. The Tempo Effects in TFMR of Chinese Women, 1970–2016

**Figure 3:**
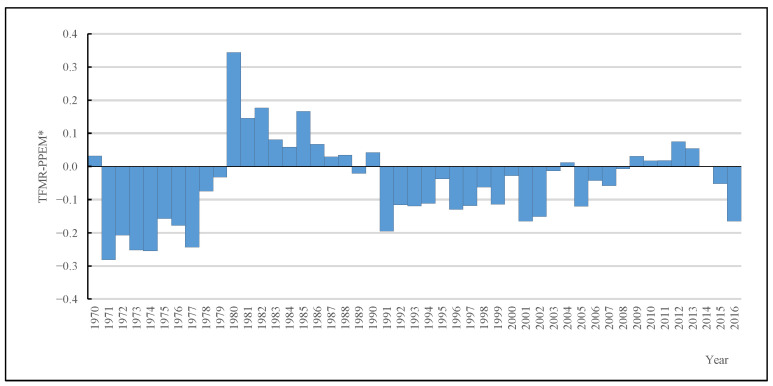
The tempo effects of Chinese women, 1970–2016. Source: 2005 China 1% Population Sample Survey and the 2017 China Fertility Sample Survey.

Figure 3 shows the trends of tempo effects in TFMR of Chinese women from 1970 to 2016. A positive or negative value represents the extent to which the TFMR is over or underestimated. We found that the TFMR is continuously affected by tempo effects. In the 1970s, the trend of tempo effects showed the waves, and the value was negative; especially from 1971 to 1977, the value of tempo effects reached −0.28 to −0.16. In these years, the TFMR was underestimated and very low. In the 1980s, the trend of the tempo effect showed a different wave pattern compared to the 1970s. It was positive and peaked at 0.34 in 1980. Afterward, the value of tempo effects gradually diminished. The TFMR in the 1980s was significantly overestimated, and its value remained above 1.00. From 1990 to 2016, except for a few years, the tempo effects were negative, but the trends did not display long-term continuous decline as in the 1980s. It changed in fluctuations and in a prolonged pattern. In 2016, the value of tempo effects reached the lowest point of −0.16 in nearly 15 years, which indicated that the tempo effect suppressed the TFMR by as much as 0.16.

### 4.3. Decomposition of Changes in Period TFMR in Four Distinct Periods, 1970–2016

The change in TFMR consists of a quantum change and tempo effects. In order to distinguish the role of the quantum change and tempo effects in Chinese women’s TFMR from 1970 to 2016, we decompose the TFMR change into its quantum and tempo components. We distinguish four distinct periods. (1) In the low and increasing period (1970–1979), the TFMR showed an upward trend but was still at a prolonged low level. (2) In the high and decreasing period (1979–1990), the TFMR was at a high level, but it began to decline after reaching its peak in 1980. (3) In the relatively stable period (1990–2007), the TFMR trends are relatively stabilized compared with the 1970–1979 and 1979–1990, and the change range of values is not large. (4) In the fluctuation and decreasing period (2007–2016), the TFMR began to decline after rising to the highest point in 2012 and then fell to the lowest in a decade in 2016. 

**Figure 4:**
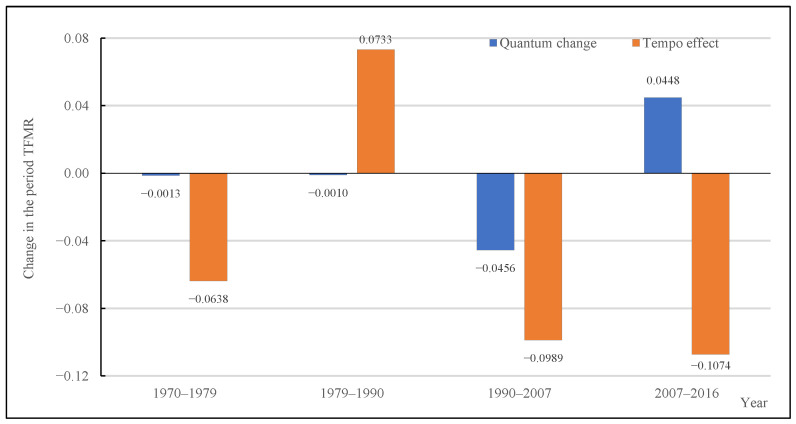
Decomposition of changes in TFMR into quantum change and tempo effect, 1970–2016. Source: 2005 China 1% Population Sample Survey and the 2017 China Fertility Sample Survey.

Figure 4 shows the decomposition of changes in TFMR into quantum change and tempo effect, and Table 1 shows the relative contribution of quantum and tempo components to changes in TFMR. Overall, the change in the TFMR was dominantly caused by the tempo effects in four periods, but the degrees are different. In the low and increasing period (1970–1979), the TFMR was at a very low level, and it declined from 1.0318 in 1970 to 0.9667 in 1979. In this period, quantum and tempo effects had identical influences on the TFMR. Still, it is mainly affected by the tempo effects, which caused the TFMR to decline by 0.0638, accounting for 98.00%. Meanwhile, the TFMR declining by 0.0013 is attributable to the quantum change. In the high and decreasing period (1979–1990), the TFMR increased by 0.0723, and the tempo effects became inverted, pushing the TFMR upward by 0.0733, and accounting for 101.38%. By contrast, the quantum change has a minimal impact, declining the TFMR only marginally by 0.0010. In the relatively stable period (1990–2007), the TFMR declined from 1.0390 to 0.8945. In this period, the tempo effects still exerted a marked role in the decline of TFMR, which led the TFMR to decline by 0.0989, accounting for 68.44%. The quantum change in TFMR change rises compared to the two previous periods. The TFMR declined, owing to the quantum change declining by 0.0456, accounting for 31.56%. In the fluctuation and decreasing period (2007–2016), the quantum and the tempo effects have contrasting influences on the TFMR. The TFMR declined from 0.8945 to 0.8319, which was dominantly due to tempo effects. The tempo effects led the TFMR to decline by 0.1074, accounting for 171.57%. Meanwhile, the quantum change increased the TFMR by 0.0448; nevertheless, the net change indicated a decline after considering the tempo effects.

## 5. Discussion

The consistent delay in the timing of first marriage and the declining TFMR of Chinese women has attracted attention from scholars and policymakers. Our study analyzed long-term trends in TFMR. Decomposition analysis was presented showing the roles of quantum change and tempo effects in the change in Chinese women’s TFMR and focusing on the period from 1970 to 2016.

Our analysis reveals that the recent decline in the TFMR in Chinese women is mainly due to the first marriage delay, not actually signaling a retreat from universal marriage. For the entire period from 1970 to 2016, the TFMR was affected mainly by tempo effects, emanating from the reasons of marriage, economic, and education policies that caused the mean age at marriage to vary. The PPEM* was employed to eliminate the tempo distortion and reflect the quantum in the total first marriage rate. The findings indicated that from 1970 to 2016, quantum change was minimal; PPEM* ranged between 0.95 and 1.00 each year. This shows a universal marriage (first marriage) among Chinese women, just the question of delayed first marriage timing.

The analysis also decomposed changes in TFMR into quantum and tempo components in four distinct periods, 1970–1979, 1979–1990, 1990–2007, and 2007–2016. The results revealed that the change in the TFMR was dominantly due to the tempo effects which were caused by the changing first marriage timing in these four periods. The TFMR declined in 1970–1979, 1990–2007, and 2007–2016. While the decline was all linked to the increase in women’s mean age at first marriage (caused by delayed timing of first marriage), varied factors caused the delay in each period. The TFMR decline from 1970 to 1979, which was at a low level, was mainly due to tempo effects, which were connected to the “later marriage” policy. The policy encouraged young couples to delay marriage until 25.00 and 23.00 years for males and females, respectively, which was a departure from the 20.00 and 18.00 years in the 1950 marriage law. The increase in the mean age at first marriage, which caused a decline in the TFMR during 1990–2007 and 2007–2016, was primarily due to economic and education expansion policies. Although the Chinese economic reform officially began in the late 1970s, it was not until 1990 before the policy started to deepen [9]. The policy manifested in the form of women’s economic participation, which made early marriage fade away as women became more financially independent, which was a status previously achievable through marriage. On the other hand, the college expansion policy introduced in 1999 greatly improved the women’s chances of attaining higher education, and an increased number of women chose to delay marriage until after completing their higher education [14]. Furthermore, higher education also brought economic value to education attainment; increased income made women more financially independent, and enlightenment from education made arranged marriages less preferred, with assortative mating taking prominence [21]. To put this in perspective, women’s mean age at marriage declined in the 1980s and began trending upwards again in the early 1990s [33]. The TFMR, which was above 1.00 in the 1980s, declined to below 1.00 in the 1990s.

In the four periods, it was only during 1979–1990 that TFMR increased. This happened because there was a reduced mean in the age at first marriage, owing to the 1981 marriage law, which relaxed the legal age requirements for marriage. In addition, China experienced a peak in birth from 1962 to 1970. The baby booms, as they are so-called, entered the marriageable age in the 1980s, thus also increasing the TFMR.

As already indicated, the value of PPEM* is a testament to the universal marriage among Chinese women. Cultural ideals sustained over time contributed to this in no small measure. Cultural ideals that have endured over time are responsible for the continued universal marriage, as demonstrated by the low quantum shift in TFMR. The Chinese culture is characterized by the Confucian ideology, which references the institution of marriage and the importance of family formation [33]. Despite the sociocultural changes in the last three decades, few people challenged the centrality of marriage [34], and the link between marriage and childbearing as a feature of Chinese family life has not really changed. Notwithstanding the growing changing attitudes toward premarital sex and cohabitation, out-of-wedlock pregnancies are more likely to lead to marriage than single-parenthood, as out-of-wedlock children in China may be denied several social benefits, including household registration [33].

Indeed, the economic growth and education expansion did elevate the status of women. The erstwhile ideology where marriage (especially early marriage) was to serve as a pathway to economic security for women has decimated. Although women may delay marriage for education or career goals, they, including the so-called “leftover women”, eventually get married [35,36].

However, scholars have argued that while universal marriage currently prevails, it is not inevitable to change in the future [37]. With the continued economic growth, education expansion, women’s economic participation and ascension to top career positions, and cross-border cultural influence, the value they derive from marriage is likely to reduce. Women, as predicted, would pay more attention to their self-development, and there would be an increased likelihood of women further postponing marriage (first marriage) or giving it up completely. In the study by Feng [37], the proportion of never-marrying in the 1980 birth cohort of Chinese women is predicted to range between 1.48% and 6.39%, and it may go up for subsequent cohorts. Where a cohort’s never-married prevalence is up to 5%, it is not regarded as universal marriage [13]. This prediction would be an unparalleled break from China’s centuries-old history of universal female marriage.

## 6. Conclusions

Given that tempo distortion caused by marriage postponement influence period indicators, our study presented a decomposition analysis showing the roles of quantum change and tempo effects in the change in Chinese women’s TFMR from 1970 to 2016. The finding suggests that the recent decline in TFMR among Chinese women is subject to first marriage delay and not a departure from universal marriage. Generally, marriage postponement leads to childbirth delay, especially in the Chinese culture, where childbearing and rearing are expected to happen only within the context of marriage. As marriage delays contribute to fertility decline [38], it is vital to correctly assess the first marriage level among Chinese women to determine the development and sustainability of the population structure. While our study has attempted to measure the quantum change and tempo effects of change in TFMR, future studies are also welcome to continue to improve on removing the tempo distortion of period indicators to accurately measure the first marriage level and trends.

## Figures and Tables

**Table 1:** Contribution of quantum and tempo components to changes in TFMR, 1970–2016.

Period	TFMR at the Start of the Period	TFMR at the End of the Period	Difference	Quantum	Tempo
1970–1979	1.0318	0.9667	−0.0651	−0.0013	−0.0638
				(2.00%)	(98.00%)
1979–1990	0.9667	1.0390	0.0723	−0.0010	0.0733
				(−1.38%)	(101.38%)
1990–2007	1.0390	0.8945	−0.1445	−0.0456	−0.0989
				(31.56%)	(68.44%)
2007–2016	0.8945	0.8319	−0.0626	0.0448	−0.1074
				(−71.57%)	(171.57%)

Source: 2005 China 1% Population Sample Survey and the 2017 China Fertility Sample Survey.

## Data Availability

The datasets used and/or analyzed during the current study are available from the corresponding author on reasonable request.
